# The German version of the Bergen Yale Sex Addiction Scale (BYSAS): psychometric properties and initial steps of validation

**DOI:** 10.1186/s40359-025-02445-1

**Published:** 2025-02-10

**Authors:** Michaela Hiebler, Hannah Brössler, Raphael Wimmer, Zoe Zipper, Elisa Renner, Jasmin Brouschek, Jürgen Fuchshuber, Aljoscha Neubauer, Human-Friedrich Unterrainer

**Affiliations:** 1 Center for Integrative Addiction Research, Grüner Kreis Society, Vienna, Austria; 2 Department of Psychology, University of Graz, Graz, Austria; 3 Faculty of Psychology, Sigmund Freud University Vienna, Vienna, Austria; 4 Faculty of Psychotherapy Science, Sigmund Freud University Vienna, Vienna, Austria; 5 Department of Psychoanalysis and Psychotherapy, Medical University Vienna, Vienna, Austria; 6 Comprehensive Center for Clinical Neurosciences and Mental Health, Medical University Vienna, Vienna, Austria; 7 Department of Religious Studies, University of Vienna, Vienna, Austria; 8 Department of Psychiatry and Psychotherapeutic Medicine, Medical University of Graz, Graz, Austria

**Keywords:** Test Adaptation, Hypersexuality, Sex addiction, Psychiatric symptoms, German version

## Abstract

**Objectives:**

This study presents the German version of the Bergen Yale Sex Addiction Scale (BYSAS). The questionnaire screens for different risk levels of problematic excessive sexual behavior (“hypersexuality”).

**Methods:**

Based on an online sample (*N* = 492), a confirmatory factor analysis as well as other reliability analysis were conducted. Demographic characteristics of the sample were also assessed. The BYSAS was validated with psychiatric symptoms.

**Results:**

Global fit statistics indicate a one-factorial structure of the BYSAS. Cronbach’s *α* was 0.77. 2.03% of the study participants were categorized as “high risk” regarding sex addiction.

**Discussion:**

Initial results suggest that the German version of the BYSAS is a promising screening instrument for sex addiction. However, further validation in clinical populations is essential to ensure generalizability and clinical utility. In addition, different risk levels as well as the mechanisms underlying hypersexuality need to be examined more closely.

**Supplementary Information:**

The online version contains supplementary material available at 10.1186/s40359-025-02445-1.

## Introduction

While problematic excessive sexual behavior (“hypersexuality”) has often been discussed as a form of behavioral addiction, there is still some controversy regarding the operationalization of this concept. In recent years, the Bergen–Yale Sex Addiction Scale BYSAS [3] has been introduced as a reliable method for assessing hypersexuality based on established components of addiction (i.e., tolerance, mood modification, salience/craving, relapse/loss of control, conflict/problems, and withdrawal). To increase the applicability of this questionnaire, this study aimed to generate a German version of the BYSAS.

Hypersexuality as a kind of addictive disease is a relatively recent phenomenon, characterized by various competing perspectives on its terminology [45], degrees of severity [34], etiology, prevalence estimates, classification, measurement and a lack of a shared definition [37]. Often mentioned symptoms of hypersexuality include an experienced loss of control over sexual stimuli and actions and thus, negative consequences like personal distress e.g. [21]. However, some researchers doubt whether hypersexuality is a disorder at all, as research on hypersexuality is often “marked by an essential social conservatism” ([33], p. 17). Correspondingly, the International Statistical Classification of Diseases and Related Health Problems (11th ed.) classifies hypersexuality as a “Compulsive sexual behavioral disorder” within the “Impulse control disorders” [46], whereas the Diagnostic and Statistical Manual of Mental Disorders (5th ed.) has not included hypersexuality in any manner in its latest edition [1]. Therefore, there is still no consensus on whether hypersexuality is a behavioral addiction e.g., [8, 18] or a disorder within the impulsive/compulsive spectrum e.g., [11]. However, there are several reasons why hypersexuality is most comparable to behavioral addictions. For example, positive feelings like excitement dominate within the anticipation phase [8]. Furthermore, symptoms like decreasing sensitivity to sexual behavior and stimuli over time [18], as well as “craving”, “abstinence” and “dependency” ([37], p. 97) align with those typical for a behavioral addiction.

To date, only a few screening instruments exist to measure hypersexual tendencies independent of sexual activities (e.g., fantasies, masturbation, sexual intercourse, watching pornography) and media (e.g., cybersex, telephone sex), especially for the German-speaking population. German-speaking studies to date mostly focused only on specific behaviors like online-sex [5], cybersex [25] or watching porn [4]; or classified hypersexuality as a compulsive disorder [6, 24]. Furthermore, while the German version of the Hypersexual Behavior Inventory [23] shows good psychometric properties, the scale may strongly align with sociocultural norms (e.g., Item 10: “I do things sexually that are against my values and beliefs.”, Reid et al. [35], p. 36). Globally, many scales are also often content specific (e.g., only focused on “Porn Addiction”; Ergün [15]). Other measurements may lack construct validity. For example, the 45-items Sexual Addiction Screening Test (SAST-R; [9]) also assesses paraphilic behavior, experiences of sexual abuse, sex work and casual online dating activity; issues, which are not inevitably part of hypersexuality. Other scales only provide a dichotomous response format e.g., PATHOS [10]. The BYSAS overcomes these methodological shortcomings through its applicability for the general population, the inclusion of sexual experiences independent of sexual activities and media, its shortness and its differentiated response format. Hence, this study aims to validate the Bergen Yale Sex Addiction Scale (BYSAS; [3]) to test its application for a German-speaking population.

There are some demographic aspects, which showed associations with hypersexuality in previous research. Therefore, possible societal differences within the BYSAS will be analyzed. For example, there is a substantial dependence on gender, as men seem to show hypersexual tendencies more often than women (e.g., [37, 44]). However, as McKeague [27] argues, studies regarding hypersexuality appear to be male-biased. Furthermore, men may seek help more frequently than women due to the heightened shame and social stigma associated with women being sexually active [27]. Furthermore, age seems to be negatively associated with hypersexuality e.g., [41]. Higher BYSAS scores also appear to be more prevalent among those who are single and with a higher education [3]. In addition to connections with demographic aspects, hypersexuality appears to be highly comorbid, with affective and anxiety disorders being the most common comorbid disorders [32, 38]. Accordingly, hypersexual tendencies appear to be present in every fourth individual diagnosed with a mood or anxiety disorder [29].

Therefore, this study aims to develop a German version of the BYSAS [3] and to assess connections with common psychiatric symptoms. The focus is on assessing the internal consistency and factor structure of the German version of the BYSAS. Generally, similar results as observed in previous validation and translation studies are expected, including acceptable internal consistency and model fit regarding a one-factor structure [3, 31, 41, 47, 48]. Furthermore, based on prior studies, we hypothesize that men will score higher on the BYSAS than women e.g., [37, 44], that single individuals will score higher than partnered individuals e.g., [3, 41], and that the BYSYS score correlates negatively with age e.g., [41].

## Materials and methods

### Sample and procedure

Initially, 947 individuals participated in this study. Data of participants who did not complete the full study (*n* = 451) was not analyzed. In detail, 31% started the survey but did not answer any questions which indicates that they were not a good match for the scope and the goals of the study and therefore decided not to participate. Furthermore, 17% terminated their participation before reaching the last page of the survey which indicates that they were interrupted during the participation or changed their mind.

There were no exclusion criteria other than German level < C1 and age < 18 years. Accordingly, three participants were excluded due to a lower German level; one participant was excluded because of an implausible age. The data collection was conducted on a Lime Survey^®^ platform after informed consent was obtained. The survey included a variety of demographic questions and standardized questionnaire measurements described below. As the online survey was set to “forced responding”, there was no missing data for participants that completed the full study. The online sample was recruited via social media channels and online platforms. The study was carried out in accordance with the Declaration of Helsinki. The studies, which involved human participants were reviewed and approved by the University of Graz (GZ. 39/5/63 ex 2023/24) and the SFU Vienna (HD65DXEGC54CWD90938), Austria. Participants gave informed consent to participate in this study. The research did not receive any specific grant from funding agencies in the public, commercial, or not-for-profit sectors.

## Psychometric assessment

### Bergen yale sex addiction scale

The *Bergen Yale Sex Addiction Scale* BYSAS; [3] is a screening instrument assessing different risk levels for sexual addiction within the general population. It consists of six items with each item representing core addiction criteria: “Salience”, “Tolerance”, “Mood Modification”, “Relapse”, “Withdrawal”, “Problems” (see *Supplementary Materials*, Table 5). Internal consistencies for the BYSAS range from α = 0.78 [3, 48] to α = 0.88 [47].

The German version of the BYSAS was created by authors of this article. First, the English items were translated into German by a bilingual person whose first language is the target language and whose cultural background is that of the target population. The response format and the polarity of the items from the English-language original version were retained.

Then the items were back-translated by a bilingual person, outside this research group and totally blind to the original version, whose first language is the original language. This back-translation was compared to the original version, finding only minor deviations. These deviations were discussed and addressed especially regarding cultural adaptation until a consensus was reached, involving all individuals that took part in the translation process as well as the lead investigators of this study that have extensive experience in the cross-cultural validation of psychological and psychiatric questionnaires.

A 5-point Likert scale (0 = very rarely, 1 = rarely, 2 = sometimes, 3 = often, and 4 = very often) was used as a response format. The time frame concerned the past year. Based on Andreassen et al. [3], four different risk groups were differentiated: A total score of 0 was defined as “no sex addiction”. A total score from 1 to 6 was defined as “low sex addiction risk” as maximally two of the six items could have a score above cut-off. Having a total score of 7 or above, but not fulfilling the full criteria for sex addiction, was defined as a “moderate sex addiction risk” as this implies a mean score above 1 on all items.

To be classified as a “sex addict” according to the BYSAS, more than half of the assessed symptoms had to be present at a specific magnitude (i.e., scoring at least 3 = often or 4 = very often) in line with the operationalization of cut-offs for other questionnaires assessing behavioral addictions e.g., [2, 26]. Consequently, individuals who scored at least 3 (often) for at least 4 times were classified as having a “high sex addiction risk”.

### Brief symptom inventory

The *Brief Symptom Inventory* (BSI-18; Franke [17]) is a widely used questionnaire to measure psychopathological symptoms of the last seven days. The resulting Global Severity Index (GSI) is based on the three subscales “Depression”, “Anxiety” and “Somatization”. A 5-point Likert scale is used in the response format differentiating from (0) not at all to (4) very strongly. Internal consistency varies from α = 0.63 to 0.93 [42].

### Statistical analysis

For the following analysis, the platform Jamovi^®^ (Version 2.5) was used. To assess the required sample size for confirmatory factor analysis, we used a sample size calculator [22], estimating average factor loadings of 0.6 and average factor correlations of 0.3. This resulted in a suggested sample size of 274. A confirmatory factor analysis (CFA) was conducted to explore the underlying factorial structure of the BYSAS. The scale is theoretically grounded on a single factor, which has been supported by previous validation studies. Therefore, it was anticipated that the analysis would confirm a one-factor structure underlying the BYSAS. Model cut-offs were used to assess the fit of underlying data for theoretical validation: a ratio < 2 between X² and df [43], a Comparative Fit Index (CFI): > 0.95 [39], and finally a Root Mean Square Error of Approximation (RMSEA): < 0.05 [7].

Cronbach’s α was analyzed to investigate the BYSAS’ reliability. An acceptable value of at least 0.7 was defined as a cut-off [13]. Discriminatory power, the scale’s reliability without a specific item (1–6) and intercorrelations between the BYSAS items, were calculated. Additional characteristics of each item and the total score were analyzed to investigate the participant’s response behavior.

Furthermore, analyses were conducted to compare the four different risk groups. To test for demographic differences regarding gender and relationship status, non-parametric analyses were chosen due to large range in group sizes. Pearson correlation analyses were used to examine the connections between study variables.

## Results

### Sample characteristics

Overall, the data of 492 individuals was analyzed (see Table 1). Participants identified predominantly as women (74%) and were on average 28 years old (*SD* = 12 years). Biological sex of the participants was not evaluated in the study. Most participants were born in German-speaking countries (AT, DE, CH; 92%). The majority was single (54%) and had at least a high-school diploma (60%).

**Table 1:** Sample characteristics

Variable		*N* = 492	% = 100
Gender	Women	363	73.78
	Men	112	22.76
	Diverse	17	3.46
Nationality	AT, DE, CH	451	91.67
	Other EU	27	5.49
	Non-EU	14	2.85
Relationship	Single	267	54.27
	In relationship	164	33.34
	Married	51	10.37
	Divorced	10	2.03
Education	High-school diploma	296	60.16
	Bachelor degree	108	21.95
	Master degree	67	13.62
	Apprenticeship	29	5.89
	Doctoral degree	8	1.63
	Other university degree	7	1.42

*Nationality*: *AT *Austria, *DE *Germany, *CH *Switzerland, *EU *European Union

### Confirmatory factor analysis

For analyzing the underlying factor-structure of the BYSAS, a Confirmatory Factor Analysis (CFA) was conducted, in which the loadings of the six items on the latent factor “sex addiction risk” were analyzed. Every item loaded on the criterion, ranging from *ß* = 0.49 (*p* = < 0.001; Item 6) to *ß* = 0.75 (*p* < .001; Item 2). This model showed a poor fit (X² = 199, *p* < .001; CFI = 0.77; RMSEA = 0.21 ,95 CI [0.18; 0.23]).

**Fig. 1:**
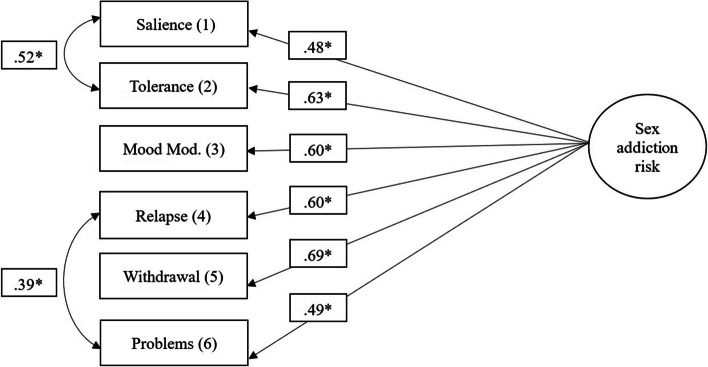
Confirmatory Factor Loadings Notes: *N*=492, * = *p* <.01, Mood Mod. = Mood Modifaction

Due to high residuals for observed correlation matrix between Item 1 and Item 2 as well as Item 4 and Item 6, post-hoc modification indices were used to add the correlated errors to the model. This allowance of free covariances between the variable pairs lead to a considerably better fitting model (X² = 10.5, *p* = .016; CFI = 1.00; RMSEA = 0.03, 95 CI [0.00; 0.07]; see Fig. 1). Residual covariances varied between *r* = .39 (*p* < .05; Item 4 and Item 6) and *r* = .52 (*p* < .05; Item 1 and Item 2). Furthermore, standardized item loadings on the criterion ranged from *ß* = 0.48 (*p* < .05; Item 1) to ß = 0.69 (*p* < .05; Item 5; see Fig. 1).

### Reliability and descriptive parameters

The internal consistency of the BYSAS was acceptable (*α* = 0.77). Discriminatory power ranged between *r* = .56 (Item 6) to *r* = .79 (Item 2). Moreover, the exclusion of any BYSAS item would invariably have compromised the internal consistency (see Table 2). Table 3 shows the BYSAS items intercorrelation matrix. The lowest correlation (*r* = .19, *p* > .01) was found between Item 1 and Item 6, the highest correlation was *r* = .66 (*p* > .01; Item 1 and Item 2).

**Table 2:** Descriptive characteristics and Reliability Statistics of BYSAS items and total score

Variable	Discriminatory power	α if item is dropped	M	SD	Mdn	Mode	IQR	Skew	Kurt	SHAP
Total score			5.49	3.82	5.00	4.00	5.00	0.9	0.84	0.94**
Salience (1)	0.53	0.74	2.03	1.08	2.00	2.00	2.00	− 0.18	− 0.46	0.91**
Tolerance (2)	0.64	0.7	1.46	1.06	1.00	1.00	1.00	0.21	− 0.83	0.9**
Mood Mod. (3)	0.5	0.75	0.88	1.12	0.00	0.00	2.00	1.03	− 0.02	0.77**
Relapse (4)	0.52	0.74	0.32	0.72	0.00	0.00	0.00	2.57	6.81	0.51**
Withdrawal (5)	0.55	0.73	0.6	0.91	0.00	0.00	1.00	1.36	0.86	0.69**
Problems (6)	0.45	0.76	0.2	0.59	0.00	0.00	0.00	3.34	11.59	0.38**

*N* = 492, ** = *p* < .01, *Mood Mod*. Mood Modification, Likert scale: 0 = very rarely 1 = rarely 2 = sometimes 3 = often 4 = very often, Total score = Sum of items 1-6, Discriminatory power = Item-total correlation, *M *Mean, *SD *Standard deviation, *Mdn *Median, *IQR *Interquartile range, *Skew *Skewness, *Kurt *Kurtosis, *SHAP *Shapiro-Wilk Test for BYSAS total score and BYSAS items (1-6)

**Table 3:** Intercorrelations of BYSAS items and total score with BSI scores

Variable		1	2	3	4	5	6	7	8	9	10	11
BYSAS	Salience (1)	-										
	Tolerance (2)	0.66**	-									
	Mood Mod. (3)	0.32**	0.4**	-								
	Relapse (4)	0.24**	0.35**	0.37**	-							
	Withdrawal (5)	0.35**	0.44**	0.38**	0.42**	-						
	Problems (6)	0.19**	0.25**	0.33**	0.56**	0.35**	-					
	Total score	0.71**	0.79**	0.70**	0.65**	0.70**	0.56**	-				
BSI	Depression	0.07	0.11	0.22**	0.17**	0.08	0.17**	0.19**	-			
	Anxiety	0.09	0.13**	0.17**	0.14**	0.08	0.11	0.17**	0.67**	-		
	Somatization	0.07	0.11	0.13**	0.11	0.07	0.08	0.14**	0.53**	0.66**	-	
	GSI	0.09	0.13**	0.20**	0.16**	0.09	0.14**	0.20**	0.87**	0.90**	0.83**	-

*N* = 492, ** = *p* > .01, BYSAS items 1–6, *Mood Mod*. Mood Modification, *BSI *Brief Symptom

Inventory, *GSI *Global Severity Index

Regarding descriptive statistics (see Table 2), the average total score for participants was 5.49 (*SD* = 3.82). Individual items, mean scores ranged between 2.03 (*SD* = 1.08) for “Salience” (Item 1) and 0.2 (*SD* = 0.59) for “Problems” (Items 6). The total score as well as most items showed a normal distribution with skewness and kurtosis ranging between − 2 and 2 [19]. In line with this, the predominant answer category was “2 = sometimes” for Item 1, “1 = rarely” for Item 2 and “0 = very rarely” for all other items (see Table 2).

Regarding connections with psychopathological symptoms, higher levels of depression, anxiety and somatization as well as higher GSI scores were connected to higher BYSAS total scores (*r* = .14 to 0.20, *p* > .01; see Table 3).

### Sex addiction risk groups

Table 4 shows that the sample was predominantly located in the “low sex addiction risk” group (61.18%). The second largest group was “moderate sex addiction risk” (29.47%), followed by “no sex addiction” (7.32%). 2.03% belonged to the “high sex addiction risk” group. Descriptively, men were most frequent in the low (43.75%) or moderate (48.21%) sex addiction risk group, women (66.94%) and participants with diverse gender orientations (52.94%) were primarily represented in the low sex addiction risk group (see Table 5).

**Table 4:** Sex addiction risk levels – descriptive characteristics for gender groups

Risk groups	Men (*n* = 112; 22.76%)	Women (*n* = 363; 73.78%)	Diverse (*n* = 17; 3.46%)	Total sample (*N* = 492; 100%)
No sex addiction	*n* = 3 (2.68%)	*n* = 30 (8.26%)	*n* = 3 (17.65%)	*n* = 36 (7.32%)
Low risk	*n* = 49 (43.75%)	*n* = 243 (66.94%)	*n* = 9 (52.94%)	*n* = 301 (61.18%)
Moderate risk	*n* = 54 (48.21%)	*n* = 86 (23.69%)	*n* = 5 (29.41%)	*n* = 145 (29.47%)
High risk	*n* = 6 (5.36%)	*n* = 4 (1.1%)	*n* = 0 (0%)	*n* = 10 (2.03%)

*N* = 492, cell represents the number and % of participants within their gender group or the total sample; Risk groups were differentiated based on Andreassen et al. [3]: A total score of 0 = “no sex addiction”, a total score from 1 - 6 = “low sex addiction risk”, a total score of 7 or above (but not fulfilling the full criteria for sex addiction) = “moderate sex addiction risk”, scoring at least 3 on more than half of the items = “high sex addiction risk”

### Demographic differences in sex addiction risk

Kruskal-Wallis tests revealed a significant difference regarding gender (χ^*2*^ = 26.94, *p* < .05, ε^2^ = 0.05, d_Cohen_=0.46), with men (*Mdn* = 7) having a higher total score then women (*Mdn* = 4; *W* = −7.33, *p* < .05). Participants identifying as diverse (*Mdn* = 5) did not differ significantly from men (*W* = −2.44, *p* < .197) or women (*W* = 0.73, *p* = .862). Furthermore, Pearson’s *r* revealed a negative correlation between the BYSAS total score and age (*r* = − .13, *p* < .05). Lastly, the BYSAS total score did not differ between forms of relationship status (Kruskal-Wallis: *χ*^2^ = 6.66, *p* < .082, ε^2^ = 0.01).

## Discussion

The purpose of this study was to validate the Bergen Yale Sex Addiction Scale (BYSAS) for German-speaking countries. In terms of the results of the CFA, every item loaded on the latent factor, thus this is in line with the assumed underlying one-factorial structure. However, the highest indicator was only *ß* = 0.69 (“Withdrawal”, Item 5). Considering residual covariances, it must be suspected that there are relationships between two pairs of indicators (Item 1 and Item 2, Item 4 and Item 6) that cannot be fully explained by the latent variable *sex addiction risk*. Accordingly, the Norwegian (*r* = .54) and the Italian (*r* = .46) version presented similar residuals between Item 1 and Item 2, indicating a lack of local independence [3, 41]. In detail, considering the content of these items, the residual correlation is likely not primarily due to logical consistency, but potentially reflects a motivational overlap, in that salience could be a factor leading to increased sex urge [3]. A similar motivational overlap might be present in Item 4 and Item 6, with the perceived negative impact leading to a desire to cut down.

As statistical parameters demonstrated, a strong general central tendency and low differentiation in “Relapse” (Item 4) and “Problems” (Item 6) may have lowered the questionnaire’s informativeness. Consistently, Zarate et al. [48] also found “Problems” (Item 6) to reveal little test information. Hence, they argue that a higher score in “Salience” (Item 1) and “Tolerance” (Item 2) may describe symptoms more common, normalized or socially acceptable, whereas “Relapse” (Item 4) and “Problems” (Item 6) seem to be applicable only for people suffering from more severe hypersexuality [48].

In terms of reliability, the internal consistency of *α* = 0.77 was lower than the one in the original version (*α* = 0.83; [3]), but still comparable with other BYSAS studies ranging from *α* = 0.78 to 0.79 [31, 41, 48]. Furthermore, intercorrelations of the BYSAS items differed highly. Those results are also mirrored by previous studies [3, 31, 41, 47, 48].

In this study, 2.03% of the participants were classified as having a “high sex addiction risk”. This rate is higher than in the original BYSAS study (Andreassen et al. [3]; “high sex addiction risk”: 0.74%, *n* = 23533). The comparability with other studies using the BYSAS is complicated as some used different cut-off scores for the risk groups. For example, Paz et al. [31] classified every participant scoring 18 or above as a being at “high risk” (3.95%, *N* = 177) and [48] used as score above 20 to define “high risk” (1,8%, *N* = 968). Furthermore, Soraci et al. [41] and Youseflu et al. [47] did not use any classification at all. Globally, prevalence estimations are detected up to 8.6% [14].

Concerning demographic differences, men had a higher total score than women and diverse participants. Corresponding differences have also been shown in other studies using the BYSAS [3, 41, 48]. However, while the medium effect size found in this study underscores the importance of exploring the effects of gender in hypersexuality, more research on possible gender differences is needed. To date no clear clinical implications can be determined. This is underlined by studies indicating different item functioning for men versus women concerning the BYSAS [3, 48]. To the best of the authors’ knowledge, this is the first BYSAS validation study to also integrate diverse gender orientations in the analyses. However, further studies including more individuals of this group are needed to better explore hypersexuality in individuals with diverse gender orientations.

In accordance with other studies [3, 41], the association between sex addiction risk and a younger age was confirmed. Correspondingly, Beutel et al. [5] also observed age differences regarding online hypersexual behavior within a German sample. Yet, a higher age does not necessarily serve as a buffer against the development of sexual addiction, as Sevcikova et al. [40] showed in their study investigating in online sex addiction tendencies within a sample aged at least 50.

Contrary to the findings of Andreassen et al. [3, 41], no variations in sex addiction risk related to relationship status were detected in this study as well as the study by Zarate et al. [48]. When tested specifically within the context of cybersex/online sex, hypersexuality also does not seem to differ according to relationship status [12, 40]. Therefore, a possible connection between relationship status and hypersexuality needs further exploration, that should also include connected variables such as relationship satisfaction, relationship duration or attachment style.

Regarding psychiatric symptoms, positive associations between sex addiction risk and every BSI-18 subscale were found. Especially the links between hypersexuality, depression and anxiety strongly resonated with previous research e.g., [16, 20, 38].

### Limitations and perspectives for future research

Restrictively, the sample of this study was not balanced in terms of gender or other demographic aspects. Regarding sample size, the approach taken in this study is one of many possible approaches. For example, following the guidelines form Muthén and Muthén [28] is also often recommended. Furthermore, biological sex of the study participants was not evaluated. Especially the overrepresentation of women in the sample likely skews the results. Therefore, future studies might profit from an approach that more clearly targets men and individuals with other gender orientations, e.g., by promoting studies on specific social media channels or websites and addressing the relevance of the study goals for these groups. Moreover, a larger sample size would have been preferable, especially given the online nature of the study. Several studies on the advantages and disadvantages of online-surveys have shown that online-surveys lead to similar response quality compared to traditional paper and pencil surveys [30]. Furthermore, online-surveys appear to lower levels of social desirability responding. While biases may occur in web surveys due to self-selection, under-coverage, non-response, online-surveys are more likely to reach more divers participants. Lastly, a major issue in online-surveys is the participation rate, with several studies reporting that only 40% or less of participants submitted completed forms [30]. Future clinical research is also needed to more closely examine individuals with “high sex addiction risk” across various genders, ages, and sexual orientations to further investigate underlying psychopathological and personality aspects as well as potential treatment effects.

In addition, more research is needed to be able to better interpret the BYSAS total score and to better assess different risk levels. To date, studies have reported different mean total scores for the BYSAS with this study reporting a mean of 5.49 (*SD* = 3.82), compared to 7.61 (*SD* = 4.8) in Soraci et al. [41] and 3.54 (*SD* = 4.14) in Andreassen et al. [3]. Additionally, the “low” and “moderate” risk groups in this study were larger than the “no risk” group. In line with this, “0 = very rarely” was not the predominant answer category for every item. Hence, the response behavior observed in this study raises further questions about how these risk categories are defined and differentiated. Further studies including individuals diagnosed with compulsive sexual behavior disorder according to ICD 11 will be needed to gain a better understanding of the predictive value of BYSAS risk groups.

In line with this, while only a small number of participants in this study (7.32%) fell into the category “no sex addiction” this does not imply a high prevalence of hypersexuality. On the contrary, it is important to avoid over-pathologizing the population. Consequently, the BYSAS should be applied solely as a screening instrument. Clinicians should comprise detailed biopsychosocial anamnesis, which consider patients on a highly individual level [45]. Accordingly, distinguishing between pathological hypersexual behavior, involving suffering and a loss of control, from healthy, highly sexual behavior should be crucial for future research [33, 36].

In conclusion, the German version of the BYSAS is a psychometrically promising and short screening instrument applicable for the general population. Future studies are needed to better understand hypersexuality and connected parameters in psychiatric and healthy populations.

## Supplementary Information

Additional file 1.

## Data Availability

The data that support the findings of this study are not openly available due to reasons of sensitivity and are available from the corresponding author upon reasonable request. Data are located in controlled access data storage at University of Vienna.
