# Supplementary material for: The German version of the Bergen Yale Sex Addiction Scale (BYSAS): psychometric properties and initial steps of validation
Source: BMC Psychol. 2025 Feb 10;13:109. doi: 10.1186/s40359-025-02445-1 (PMC11812235; doi:10.1186/s40359-025-02445-1)
Supplement: Supplementary file 1 — Additional file 1. [file 40359_2025_2445_MOESM1_ESM.docx]

**Supplementary material**

*Bergen Yale Sex Addiction Scale (BYSAS); translated from the German version*

| **Instruction** | | |
| --- | --- | --- |
| The following questions are related to your sexual activities. This refers to various sexual fantasies, desires and behaviors, e.g. masturbation, pornography consumption, sexual activities with consenting adults, cybersex, telephone sex, strip clubs, prostitution and the like. Choose the one that best describes you from the answer alternatives. Again, rest assured that your data will be treated strictly confidential and anonymous. | | |
| **Items** | | |
| No. | Item | *How many times within the last 12 months...* |
| 1 | Salience | *... have you spent a lot of time thinking about sexual activity?* |
| 2 | Level of tolerance | *... have you felt the urge to engage in sexual activity more and more often?* |
| 3 | Mood modification | *... have you used sexual activity to forget personal problems or escape them?* |
| 4 | Relapse / Loss of control | *... have you tried to limit the extent of your sexual activity without success?* |
| 5 | Withdrawal symptoms | *... have you been restless when you were denied your sexual activities, e.g. when there was no opportunity to do so?* |
| 6 | Conflicts / Problems | *... have you been so sexually active that it has had a negative impact on your life (your private relationships, your household, your health, your job/studies)?* |
| **Response format** | | |
| 0 = Very rare, 1 = Rare, 2 = Sometimes, 3 = Often, 4 = Very often | | |
